# Deep Learning in the Recognition of Activities of Daily Living Using Smartwatch Data

**DOI:** 10.3390/s23177493

**Published:** 2023-08-29

**Authors:** Ariany F. Cavalcante, Victor H. de L. Kunst, Thiago de M. Chaves, Júlia D. T. de Souza, Isabela M. Ribeiro, Jonysberg P. Quintino, Fabio Q. B. da Silva, André L. M. Santos, Veronica Teichrieb, Alana Elza F. da Gama

**Affiliations:** 1 Centro de Informática, Universidade Federal de Pernambuco, Recife 50740-560, PE, Brazil; afc8@cin.ufpe.br (A.F.C.); vhlk@cin.ufpe.br (V.H.d.L.K.); tmc2@cin.ufpe.br (T.d.M.C.); jdts@cin.ufpe.br (J.D.T.d.S.); imr@cin.ufpe.br (I.M.R.); fabio@cin.ufpe.br (F.Q.B.d.S.); alms@cin.ufpe.br (A.L.M.S.); vt@cin.ufpe.br (V.T.); 2 Projeto CIn-UFPE Samsung, Centro de Informática, Recife 50740-560, PE, Brazil; jpq@cin.ufpe.br; 3 Departamento de Engenharia Biomédica, Universidade Federal de Pernambuco, Recife 50740-560, PE, Brazil

**Keywords:** human activity recognition, neural networks, smartwatch, wearable sensor data

## Abstract

The recognition of human activities (HAR) using wearable device data, such as smartwatches, has gained significant attention in the field of computer science due to its potential to provide insights into individuals’ daily activities. This article aims to conduct a comparative study of deep learning techniques for recognizing activities of daily living (ADL). A mapping of HAR techniques was performed, and three techniques were selected for evaluation, along with a dataset. Experiments were conducted using the selected techniques to assess their performance in ADL recognition, employing standardized evaluation metrics, such as accuracy, precision, recall, and F1-score. Among the evaluated techniques, the DeepConvLSTM architecture, consisting of recurrent convolutional layers and a single LSTM layer, achieved the most promising results. These findings suggest that software applications utilizing this architecture can assist smartwatch users in understanding their movement routines more quickly and accurately.

## 1. Introduction

Adhering to the World Health Organization’s (WHO) recommended levels of physical activity can prevent and help combat coronary heart disease, hypertension, and diabetes, as well as reducing the risk of developing cancer and the symptoms of depression and anxiety [1]. However, many people are unaware of the negative consequences associated with leading inactive lives, often failing to recognize the potential harm they inflict upon themselves, as sedentary lifestyles have become increasingly prevalent in the past few decades [2], significantly impacting both the physical and mental health of individuals.

To address this issue, wearable devices have emerged as powerful tools in monitoring and improving the overall health of individuals [3,4]. These devices, especially smartwatches, equipped with advanced sensors, offer insights into our daily activities, providing a deeper understanding of our routines and self-care practices. By wearing these devices regularly, people can gain a heightened awareness of their physical exertion levels and make informed decisions to incorporate more active behaviors into their daily lives [3]. Smartwatches are primarily studied for monitoring or promoting physical activity in patients with diverse health conditions. However, their utility extends to other activities, including assessing sleep, monitoring heart rate, managing diabetes, and aiding in dietary tracking [4,5].

Wearable devices provide readily available data that are frequently utilized to provide feedback based on activity measurements [4], enabling users to achieve their exercise goals as quantified by these devices. Smartwatches play a vital role in collecting essential data, generating information that can facilitate users in modifying their daily movement levels. Researchers, like Cheatham et al. [3], have demonstrated the benefits of using smartwatches for individuals engaged in physical activity with weight loss objectives. In this pursuit of effectively assisting users in self monitoring, some studies center on the recognition of human activities through data collected by wearable devices, such as smartwatches and smartphones [6].

Sensor data serve multiple purposes, exemplified by their application during an outdoor run. In this scenario, the smartwatch not only identifies the specific activity being performed but also provides valuable insights into the distance traveled, heart rate, and rhythm distribution throughout the run. Additionally, the device can calculate the number of calories expended and assess the level of exercise intensity, aiding the runner in understanding their performance and optimizing their training goals. Notably, the primary data collected from wearable devices for activity recognition stem from accelerometers and gyroscopes, providing time-series data indexed in temporal order. This type of data enables the training of neural networks that can effectively capture the spatial and temporal context of the sensor reading sequence. To achieve this, various architectures have gained prominence, including convolutional neural networks (CNNs), long short-term memory (LSTM), and attention mechanisms [7]. Training these networks necessitates the use of relevant datasets that encompass diverse types of sensors and recognized activities, many of which have already been published [8,9,10].

The recognition of activities using deep learning can contribute to understanding a smartwatch user’s movement routine. This is mainly because an individual’s physical activity is related to all their movements during the day, which may include routine activities, such as cleaning the house, and physical exercise, such as running [11]. Furthermore, activity recognition also helps to avoid the need for manually inputting each performed activity, which can be tedious and prone to errors. With the automation of this process, users can focus more on their routines and obtain a more reliable and comprehensive record of their physical activities.

With this purpose, a review of the state-of-the-art literature on activity recognition techniques is presented in this work, along with public datasets. Building upon the research, open-source network architectures were evaluated to measure their performances in recognition of activities related to the day-to-day of an ordinary person and thus evaluate their movement routine. The findings from the evaluations of the network architectures have pointed to a particular activity recognition technique that stands out as the top performer, which positions it as the most promising method to explore further. These results allow for evaluating activity recognizers regarding their applicability to technologies aimed at understanding the physical movement routine of smartwatch users.

The remainder of this paper is organized as follows. Section 2 is related to the background on activity recognition methods, presenting the works found through the bibliographic review, highlighting their main methodological contributions, and public datasets to HAR. Section 3 describes the test methodology. Section 4 presents the selected techniques and datasets, and Section 5 shows and discusses the main results. Section 6 details the conclusions of this paper.

## 2. Background on Activity Recognition Methods and Datasets

The HAR domain, fueled by data from wearable devices, has experienced remarkable growth, driven by advancements in machine learning techniques. Various deep learning approaches have been employed to identify physical exercises and other activities in individuals’ everyday lives [12]. For instance, activities such as running, walking, climbing stairs, washing dishes, and driving can be accurately recognized. Several prominent architectures have emerged, including convolutional neural networks (CNNs), long short-term memory (LSTM), and attention mechanisms.

**Figure 1:**
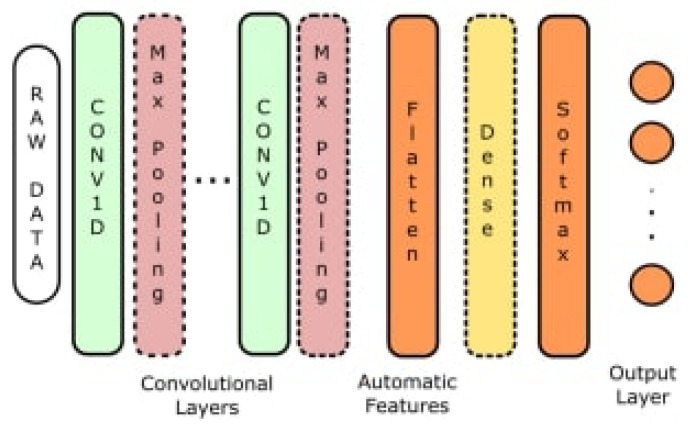
HAR architecture based on CNN [15].

CNN-based methods [13,14,15,16,17,18,19,20] have been extensively explored and recognized for various applications. CNN-based activity recognition enables fast and efficient predictions [21,22]. Cruciani [15] pre-trains a CNN using the UCI-HAR dataset [10] and optimizes the model’s hyperparameters. Additionally, the pre-trained CNN is utilized to extract features from real-world datasets, such as the Extrasensory dataset [8] as shown in Figure 1.

**Figure 2:**
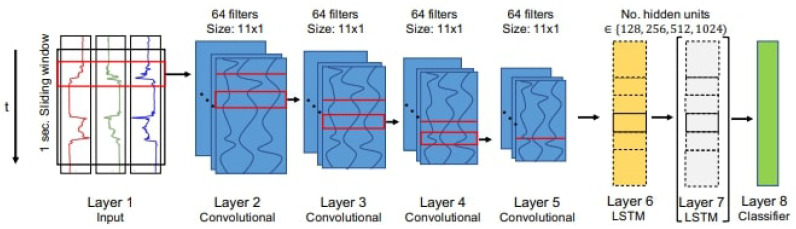
Improved DeepConvLSTM architecture [25].

In the realm of time-series analysis, LSTM has gained prominence. LSTM is a type of neural network that excels at learning from sequential data, considering temporal patterns and events that occurred earlier in time. This architecture has found popularity in activity recognition tasks [23,24,25,26,27,28]. One notable example is the DeepConvLSTM [24], which exhibits state-of-the-art results in activity classification. The performance of DeepConvLSTM has been enhanced by works that utilize a single LSTM layer instead of a two-layered LSTM [25] as it is seen in Figure 2.

Another approach involves a hybrid method [7,19,29,30,31,32] that predicts activities by extracting features from the data prior to inputting them into the neural network [7]. This technique has shown superior performance compared to various other methods evaluated using standardized benchmarks [33], encompassing datasets such as MHealth [34], USC-HAD [35], UTD-MHAD1 [36], UTD-MHAD2 [37], WHARF [38], and WISDM [39].

In most cases, training networks for activity recognition is carried out in a supervised manner, requiring the accurate labeling of various activities performed by individuals throughout the day in real time. However, there is a potential for missing annotations due to human error or perceived irrelevance. Networks like HAR-GCCN [13], a deep graph CNN model, propose leveraging the inherent chronology of human behavior to learn unknown labels. For example, bathing is expected to follow physical exercise, and this implicit chronology can be utilized with data from chronologically adjacent sensors to learn missing labels.

Additionally, unsupervised domain adaptation (UDA) techniques adapt a model trained on a source domain to a novel target domain using only unlabeled data. UDA contributes to activity recognition using wearable sensors, enhancing model training for new device users. SALIENCE [14], an unsupervised user adaptation model for multiple wearable sensors, is designed to improve activity recognition by adapting the trained model to the data patterns of new users. It incorporates an attention mechanism that emphasizes discriminating features for more accurate activity classification. Other papers utilizing attention concepts [19,20,23,25,40] for activity recognition combine various mechanisms to generate higher-dimensional feature representations used for classification, encompassing spatio-temporal feature learning and important time points.

The hierarchical network architecture [39] employs a two-level hierarchy to recognize activities initially classified as “lying down”, “sitting”, “standing in place”, “walking”, “running” and “cycling”. Furthermore, these activities are further divided into “stationary” and “non-stationary”.

Moreover, the HAR field encompasses specific datasets utilized for training neural networks. Therefore, a comprehensive mapping of relevant datasets used in these studies was conducted and is presented below.

### Datasets

Public datasets used for activity recognition exhibit distinctions, including variations in the capture methodology and the quantity and type of labels employed.

Regarding the capture methodology, datasets are predominantly obtained either in real-life scenarios or through simulations that mimic real-life conditions (e.g., [8]). Alternatively, datasets can be captured in controlled or unspecified environments (e.g., [38,41]).

The Extrasensory dataset [8] comprises over 300,000 examples (minutes) captured from 60 users in real-life scenarios. Users carried smartphones and smartwatches, which provided data from various sensors. The dataset includes annotations for activities of daily living (ADL), such as “bicycling” and “watching TV”, as well as context labels like “at school” and “phone in hand”.

HHAR (human–human activity recognition) dataset [42] was captured in projected environments that simulate realistic settings. It contains accelerometer and gyroscope data from smartphones and smartwatches worn by six participants. The dataset primarily focuses on labeling postures such as “biking”, “sitting”, “standing”, “walking”, “stair up” and “stair down”. Another dataset published by Garcia-Gonzalez [43] involves data from 19 users, collected through the smartphone accelerometer, gyroscope, magnetometer, and GPS. Participants were not constrained in terms of smartphone placement, simulating real-world scenarios. The labels in this dataset are “inactive”, “active”, “walking” and “driving”. The “inactive” label denotes activities performed without carrying the smartphone, while “active” represents activities performed using the smartphone but without significant movement. For instance, the “making dinner” activity falls under the “active” label.

The Opportunity dataset [44] is a large dataset comprising a substantial number of sensors. It includes 7 inertial measurement units, 12 3D acceleration sensors, and 4 3D localization sensors attached to the body. The capture involved four users, each performing six executions. Five of these executions simulate natural scenarios, while the sixth execution follows a predetermined sequence defined in a script, known as the “drill” execution.

The literature also includes datasets captured in controlled or unspecified scenarios. The WISDM dataset [38] captured 153 min of activity from 51 participants, resulting in 15,630,426 instances. It encompasses accelerometer and gyroscope data from smartphones and smartwatches. The dataset consists of 18 labels, including activities such as “walking”, “typing”, “brushing teeth”, “kicking” (soccer ball), and “folding clothes”. The RealWord (HAR) dataset [41] was created using 150 min of recorded data from 15 users and incorporates data from six sensor types. It features seven labels representing transitional body postures, such as going up and down stairs, jumping, lying down, standing, sitting, running, and walking. The MHEALTH dataset [34] comprises data from 10 users captured through wearable accelerometer, gyroscope, magnetometer, and electrocardiogram sensors placed on the subject’s chest, right wrist, and left ankle. The dataset includes 12 workout labels, encompassing activities such as cycling, running, and jumping forward and backward. The UTD-MAD dataset [36] utilizes a fusion of wearable inertial and depth sensors to record data. It involves eight subjects (four women and four men) performing 27 different actions. Each action was repeated approximately four times, resulting in a dataset with 861 examples. The UTD-MAD dataset [36] focuses on actions related to body movements, physical activities, and postures, including “arm cross”, “boxing” and “sit to stand”.

The UCI-HAR dataset [10] contains data from 30 subjects who performed ADL while carrying waist-mounted smartphones equipped with inertial sensors. The dataset includes six postures and transitions, with accelerometer and gyroscope data being captured. The sliding window technique with a 50% overlap was applied to the data.

The USC-HAD dataset [35] is specifically designed for ADL recognition, particularly in the healthcare domain. It consists of data from 14 individuals performing 12 activities and postures, including “walking upstairs”, “running forward” and “sleeping”.

Although other datasets [37,45,46] are also employed in activity recognition using wearable devices, this work specifically focuses on datasets utilizing data from smartphones or smartwatches. Additionally, a subset of datasets with other types of sensors was investigated, particularly those labeled for ADL.

## 3. Methodology

With the aim of conducting a comparative study on deep learning techniques for recognizing daily living activities, the primary focus was placed on the rigorous testing of three diverse techniques. Therefore, the review process encompassed the examination of 50 articles, with a predominant emphasis on 27 articles specifically addressing deep learning techniques. In parallel, a thorough evaluation encompassed the consideration of 23 datasets, ultimately culminating in the selection of 1 dataset for further investigation. This section of the article delineates the review methodology, the criteria employed for selecting the techniques and datasets, as well as the comprehensive evaluation methods utilized to assess the performance of the chosen techniques.

### 3.1. Literature Review

The exploration of techniques presented in academic papers was conducted following a scientific methodology. A comprehensive search was performed across leading research platforms, including Google Scholar, Portal Periodicos CAPES, Scientific Electronic Library Online - SciELO, and others. The snowball sampling technique was applied to identify the most-relevant papers.

The search for papers utilized specific strings such as “Human Activity Recognition”, “Human Activity Detection”, “Human Activities”, “Smartwatch”, “Smartphone”, “Wearable Sensor Data”, “Wearable Sensing”, “Wearables”, “Benchmark”, “Datasets”, “Artificial Intelligence”, “Machine Learning”, “Neural Networks”, “Deep Learning”, “RNN”, ”CNN”, “LSTM”, “Transformer” and “Self-attention”.

During the search phase, the evaluation criteria for papers included whether they employed machine learning techniques, utilized wearable device sensors, identified the type of recognized activities, provided quantitative results of activity recognition, demonstrated method originality, and had a recent publication date.

Consequently, the selected papers for cataloging were those that employed artificial intelligence for activity recognition using data from wearable devices, particularly smartphones and smartwatches. These papers demonstrated state-of-the-art results when compared to similar techniques. The catalog of papers formed the foundation for the subsequent technique selection stage, which is described in detail below.

### 3.2. Techniques Selection

Based on the cataloged information, specific criteria were employed to carefully select the techniques for an in-depth study and subsequent testing. The key requirements considered during this stage of the selection process were as follows:

- The type of sensor employed in the technique;
- The type of recognized activity;
- The availability of the repository containing the technique.

Utilizing these criteria, the chosen methods were those that demonstrated proficiency in recognizing activities related to the following:

- Activities of daily living (ADL);
- Utilization of data obtained from smartwatch sensors;
- Possession of a publicly accessible repository on the internet.

### 3.3. Dataset Selection

The selection of the dataset for conducting the technique tests was based on specific criteria that aligned with the objectives of this study and the identified datasets. The established criteria include the following:

- Public availability of the dataset;
- Inclusion of labels for ADL;
- Inclusion of data obtained from smartwatches;
- Adoption for evaluating state-of-the-art activity recognition techniques.

Once the dataset was chosen, it underwent a pre-processing stage following the standardized benchmark [33]. The details of this pre-processing procedure are described below.

### 3.4. Evaluation of Models

Pre-processing step for data standardization was performed to evaluate the models. The overlapping sliding windows technique used is described below.

#### 3.4.1. Sliding Window

**Figure 3:**
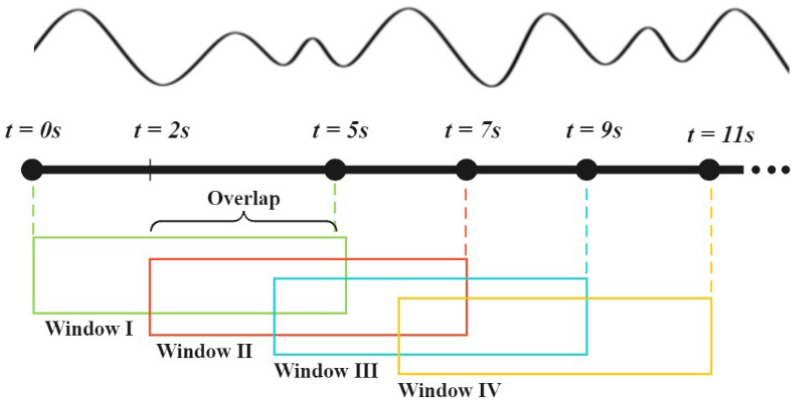
Sliding window representation.

Prior to the training or prediction process, the data undergo a sliding window technique with a 60% overlap. The fundamental concept behind the sliding window with overlap is to introduce a fixed-size window or subarray that sequentially moves over the larger dataset, allowing for operations to be performed on the data within the window. This approach facilitates the analysis and processing of the data in a systematic manner. In this particular study, the sliding window size is set to 5 s as depicted in Figure 3. A window size of 5 s offers a suitable temporal resolution for capturing temporal variations in activities [47]. Everyday activities often exhibit rapid changes and short transitions, making the utilization of a smaller sliding window more conducive to a refined temporal analysis of the data. The chosen window size facilitates the examination of activities at a more granular level, while incorporating a sufficient degree of overlap ensures the attainment of reliable statistical analyses [48]. Moreover, this approach enables the identification of local patterns and the capture of broader, enduring attributes characterizing the activities.

#### 3.4.2. Training and Evaluation

For each technique, training was conducted using the original codes available in their respective repositories. The original hyperparameters of the techniques were preserved, without implementing any fine-tuning procedures. The WISDM dataset was utilized for training, with accelerometer and gyroscope data being synchronized and pre-processed as described in the previous step.

During training, GPU and CPU usage, training time, and memory usage (both for RAM and GPU) were monitored and recorded using the Neptune tool.

Subsequently, after obtaining the trained models, various performance metrics were evaluated, including GPU usage, CPU usage, RAM usage, inference time (the time taken to predict the output), and network size (the disk space occupied by the trained model). The results for each inference metric were averaged over a thousand measurements.

To measure GPU usage and memory utilization, a parallel thread was employed, utilizing pynvml, a Python binding to the NVIDIA Management Library, to capture the percentage of processing and memory usage. For CPU usage measurement, Psutil, a cross-platform library for retrieving information on running processes and system utilization (CPU, memory, disks, network, and sensors) in Python, was used. RAM usage was measured using memory_profiler. Inference time was calculated by invoking the predict function and averaging the time using timeit. Additionally, the following metrics were also measured for each technique: accuracy, precision, recall, and F1-score.

## 4. Evaluated Techniques and Dataset

Three techniques were selected based on the criteria outlined in the Section 3. In the following subsections, we provide a detailed explanation of the evaluated techniques and the chosen dataset.

### 4.1. Selected Techniques

#### 4.1.1. Technique I

The first technique, proposed by Bock et al. [25], explores the usage of DeepConvLSTM, a deep learning architecture that combines convolutional and LSTM recurrent layers [24]. Specifically, they employ a 1-layered LSTM configuration.

The key highlights of this work are as follows:

(I) The availability of experiments in their public GitHub repository; (II) The adoption of a one-layered LSTM model, which reduces the network size and accelerates training and inference times; (III) The observation that architectures with a 2-layer LSTM outperform those using 5 popular datasets.

Bock et al. [25] conducted their experiments using datasets such as Opportunity [44], Wetlab [45], SBHAR [46], RealWorld-HAR [49], and HHAR [42], reporting maximum precision of 77.6%, recall of 76.3%, and F1-score of 74.4% using the RealWorld-HAR dataset.

#### 4.1.2. Technique II

The second selected technique is presented by Singh et al. [23]. Their approach focuses on forecasting time series data using a combination of recurrent and convolutional networks. Notably, they incorporate an attention mechanism to identify the crucial time points that contribute to the forecast.

Singh et al. [23] conduct experiments on six datasets, including MHealth [34], UTD-MHAD [36], USC-HAD [35], WHARF [37], and WISDM [38]. The results demonstrate a statistically significant advantage of their approach over other techniques, including DeepConvLSTM. They achieve accuracy values above 58% and up to 94% during training with the MHealth dataset, with recall exceeding 55% and F1-score surpassing 54% across all tests.

#### 4.1.3. Technique III

The third technique, proposed by Abdel-Salam et al. [7], involves a comprehensive literature review of human activity recognition based on wearable sensors. Additionally, they propose a hybrid neural network model that outperforms existing techniques on the MHealth [34], USC-HAD [35], and UTD-MHAD [36] datasets. Their model incorporates an independent feature extraction step followed by a neural network for activity classification using the extracted features. Abdel-Salam et al. [7] also introduce a standardized evaluation benchmark adopted in their study.

The model was initially trained on seven datasets, including MHealth, UTD-MHAD1, UTD-MHAD2, USC-HAD, WHARF, WISDM, and Opportunity. Notably, these datasets include the six datasets tested by Singh et al. [23]. Abdel-Salam et al. [7] report a mean accuracy above 70.48%, reaching 99.70% using the MHealth dataset.

### 4.2. Dataset

The chosen dataset for the experiments is the WISDM Smartphone and Smartwatch Activity and Biometrics Dataset [38], published by Weiss et al. It comprises 15,630,426 examples, which have also been utilized in other studies related to human activity recognition [7,23].

The WISDM dataset contains accelerometer and gyroscope time-series sensor data collected from smartphones (Nexus 5, Nexus 5X, and Galaxy S6) and smartwatches (LG G watch). It encompasses data from 51 test subjects, each performing 18 activities for a duration of 3 min.

Since the dataset consists of 51 participants, the data were split in such a way that one subject was allocated for validation, one for testing, and the remaining subjects for training. The splitting approach used is inspired by the leave one subject out (LOSO) method [50]. To ensure more accurate results, a total of 51 folds were created following these steps:

- The test subset starts with subject 1 and increments by 1 for each subsequent fold, utilizing subject 2, subject 3, and so on until subject 51 is reached (the last fold).
- The validation subset always consists of the subject preceding the test subject. In the case where the test subject is the first subject, the subject preceding it is considered to be the last subject (subject 51).
- The training subset consists of all subjects that are not part of the test or validation subset.

It is worth noting that the WISDM dataset has an older version [51] than the one used in these experiments. It is composed of data from six activities, collected from 29 individuals using phone-based accelerometers.

## 5. Results and Discussion

In this section, we discuss the results related to computational resources and the model’s accuracy in the conducted tests for everyday activity recognition using smartwatch sensors. Analyzing metrics such as CPU usage, GPU usage, RAM memory utilization, training and inference time, and model size is crucial when developing an application for a smartwatch.

The analysis related to model performance aims to determine the performance of these techniques in classifying ADL using accelerometer and gyroscope data directly. Additionally, the evaluation of computational resources is crucial to assess the practical feasibility of using these networks on smartwatches.

Firstly, smartwatches typically have limited computational resources, including CPU and GPU capabilities, as well as limited RAM. Monitoring these metrics is essential to ensure that the application operates efficiently within the constrained resources of the smartwatch [52]. It helps avoid excessive resource utilization, which could lead to performance issues, battery drain, or even crashes.

Secondly, the training and inference time play a vital role in real-time applications. Smartwatches often have lower processing power compared to traditional devices, making it essential to optimize the model’s performance to achieve timely results. Monitoring these metrics allows developers to identify any bottlenecks or areas for optimization, ensuring that the application can provide quick and responsive outputs.

Thirdly, the size of the model is crucial for smartwatches due to their limited storage capacity. Large models can consume a significant amount of storage space, potentially limiting the number of applications or features that can be installed on the device. Analyzing the model’s size helps developers select or design models that strike a balance between accuracy and compactness, optimizing the utilization of the smartwatch’s storage resources.

Computers used for training and inference were laptops with Intel Core i7-11800H Octa-Core processor, NVIDIA Geforce GTX 3060 Mobile graphics, 16GB DDR4 RAM, with operational system Windows 11. The experiment tracking tool used was Neptune.

The data available from Table 1 show the training metrics of each of the three techniques tested in the standardized WISDM. All inference statistics can be seen at Table 2. And Table 3 summarizes the metrics for evaluating the networks in the test suite. The accuracy, precision, recall and F-score of the test set were calculated.

### 5.1. Computational Resource Usage and Model Size

Comparing the results of Bock et al. [25] with the other two evaluated techniques, this method exhibited higher usage of RAM memory, CPU, and GPU compared to the other approaches. However, it demonstrated the lowest GPU memory usage, measuring 2.56 GB. Additionally, Bock et al.’s technique achieved the fastest training time, roughly half of Abdel-Salam et al.’s [7] time and approximately 7% of Singh et al.’s [23] time. The superior hardware usage could have contributed to the decreased training time, suggesting a faster network. During inference, it also showed the lowest time, approximately half that of the closest competitor. Moreover, it required less GPU memory, CPU, and GPU usage. However, the RAM memory usage was slightly higher than that of Singh et al. [23] and double that of Abdel-Salam et al. [7]. Additionally, the network’s storage size was larger than the other techniques. The difference in network size on disk may be influenced by the used library, as Bock et al. [25] uses PyTorch and the others use TensorFlow, each with a different method for saving the network. Despite these challenges, Bock et al.’s technique consistently outperformed the other two in almost all metrics during training and inference. It also achieved the best statistics for all metrics, including accuracy, precision, recall, and F1-score.

Evaluating the second technique, that of Singh et al. [23], it demonstrated lower values of RAM memory and CPU usage during training. However, the training time for this network was significantly longer, approximately 66 h and 18 min, which is about 14 times longer than Bock et al.’s [25] and seven times longer than Abdel-Salam et al.’s [7] training times. One possible explanation for the longer training time is that the original code was written for TensorFlow version 1 and minor modifications were made to run it on version 2, enabling a flag to disable TensorFlow eager execution, which may have impacted the speed. The GPU memory usage during training was also significantly higher for this technique. During inference, Singh et al. [23] also continued to use higher GPU memory, approximately 5.2 GB, but had similar RAM usage compared to Bock et al. [25]. Considering that the RAM memory available on a Galaxy Watch4 is 1.5 GB, all techniques had RAM usage compatible with this smartwatch.

Table 2 shows that Singh et al. [23] consistently outperformed Abdel-Salam et al. [7] in all calculated metrics, with at least 8.41% better performance during inference, based solely on quantitative evaluation of the networks.

Further memory usage tests should be performed using activity recognition applications on a smartwatch device or just the CPU, which is the processor used in smartwatches. This will enable a more accurate assessment of the feasibility of using the networks for real-time inference.

The third technique, that of Abdel-Salam et al. [7], demonstrated lower RAM, GPU, and CPU usage during training compared to that of Bock et al. [25]. However, it only showed relatively lower RAM usage during inference. In terms of other training and evaluation metrics, it did not present significant differences compared to Bock et al. [25]. Nevertheless, during network evaluation, it exhibited the worst results in all metrics.

The results so far indicate that Bock et al.’s [25] technique consistently outperforms the other two techniques in real-world applications, especially in terms of inference time and memory usage.

Regarding the size of the networks on disk, Bock et al.’s network [25] had the most compact size at 1.25 MB, which is suitable for smartwatch applications. However, a detailed feasibility analysis of applying activity recognition technology using the evaluated networks on a smartwatch requires further investigation. This topic will be a focus of future work.

### 5.2. Model Performance and Evaluation Metrics

The neural networks were evaluated using accelerometer and gyroscope data as features to classify 18 activities of daily living. The distribution of data among the activities is shown in Table 4, which was obtained after processing the raw sensor data with an ARFF header [53]. The results in Table 4 demonstrate that the class distribution across activities closely approximates the expected value of 5.55%, which was calculated by dividing 100 samples by 18 activities [53]. This balanced distribution of samples for each class contributes to a robust evaluation of the model’s performance across various activities.

The experimental results approached the maximum values found by Bock et al. [25], exhibiting an accuracy of 74.40 ± 20.40%, precision of 75.5 ± 19.70%, recall of 74.4 ± 20.40%, and F1-Score of 72.1 ± 20.90%, as shown in Table 3. These findings demonstrate the model’s robust performance in accurately classifying the activities, showcasing remarkable alignment with state-of-the-art outcomes documented in the existing literature. It emphasizes the advantageous utilization of the first technique for activity recognition in comparison to the second and third techniques.

Given these results, we can prioritize the study of the technique proposed by Bock et al. [25] for monitoring an individual’s physical movement through smartwatch data. This is of great importance to promote awareness of daily activities and encourage healthy habits. By collecting detailed information on movement patterns, the smartwatch can provide valuable insights into the amount of physical activity performed. These data can assist users in adjusting their behaviors and routines, improving their quality of life and overall well-being. Furthermore, continuous monitoring of physical movement is crucial to identify potential health issues and even detect sleep and stress patterns.

## 6. Conclusions and Future Work

In this study, we aimed to investigate the significance of activity monitoring through wearable devices in promoting individuals’ awareness of their physical activity habits. Efficient activity recognition can contribute to a more user-friendly and seamless experience, encouraging consistent usage of wearable devices for prolonged health monitoring. It also enhances the reliability of data, promoting a deeper understanding of users’ physical routines and health trends.

The study made use of wearable sensors to evaluate and compare three of the most relevant techniques for HAR. Based on the survey and evaluation of activity recognition techniques using wearable sensor data, the results highlight the effectiveness of deep learning architectures, such as convolutional neural networks (CNNs) and long short-term memory (LSTM), in accurately recognizing human activities [12]. These architectures have shown promising performance in distinguishing between different activities.

The use of WISDM, a dataset with a diverse array of activity labels, afforded the activity recognition algorithms a broad spectrum of patterns to discern, leading to excellent generalization capabilities for previously unseen data. Consequently, this opens avenues for prospective investigations concerning the expansion of novel activity types and the application of activity recognition in diverse contexts.

The findings of this study hold practical implications for the field of activity recognition and its applications in technology, such as enhancing technology development, in which the identification of the top-performing activity recognition technique, as shown in Section 5.1 and Section 5.2, provides valuable guidance for researchers and developers working on health-monitoring technologies with wearable devices.

Moreover, with a deeper understanding of users’ activity patterns, health professionals and caregivers can design personalized health interventions to address specific activity-related challenges. The study’s insights can be leveraged to provide real-time suggestions and motivational prompts based on recognized activities, as wearable devices can positively influence users’ exercise routines and foster long-term adherence to healthy habits.

It is crucial to consider the broader context of a person’s physical movement routine rather than solely focusing on specific activities. Recognizing activities beyond structured movements can provide a valuable understanding of an individual’s physical activity levels and help address sedentary behavior. This holistic understanding is essential for promoting long-term health and well-being.

Ultimately, future works and experiments in the field can explore the following:

- The optimization study of network parameters;
- Assessment of activity recognition using accelerometer-only data;
- Processing of techniques in CPU and integration with smartwatches, for evaluation of memory usage;
- Improvement in inference time.

## Figures and Tables

**Table 1:** Training metrics.

Technique	RAM Memory Usage (Mean)	GPU Memory Usage (Mean)	Training Time	CPU Usage (Mean)	GPU Usage (Mean)
Bock et al. [25]	14.43 GB	2.56 GB	4 h 38 m	50.86%	62.84%
Singh et al. [23]	11.64 GB	5.71 GB	66 h 18 m	18.47%	37.83%
Abdel-Salam et al. [7]	13.90 GB	4.48 GB	9 h 2 m	30.2%	36%

**Table 2:** Inference metrics.

Technique	RAM Memory Usage (Mean)	GPU Memory Usage (Mean)	Inference Time	CPU Usage (Mean)	GPU Usage (Mean)	Network Size (In Disk)
Bock et al. [25]	3974.6 MB	1.88 GB	1673.30 ms	6.24%	40%	1.25 MB
Singh et al. [23]	3846.4 MB	5.2 GB	3280.47 ms	7.125%	43%	398 KB
Abdel-Salam et al. [7]	1945.0 MB	4.36 GB	6006.21 ms	7.975%	47%	148 KB

**Table 3:** Neural network evaluation metrics.

Technique	Accuracy	Precision	Recall	F1-Score
Bock et al. [25]	0.744 ± 0.204	0.755 ± 0.197	0.744 ± 0.204	0.721 ± 0.209
Singh et al. [23]	0.623 ± 0.185	0.634 ± 0.180	0.623 ± 0.185	0.606 ± 0.187
Abdel-Salam et al. [7]	0.580 ± 0.244	0.580 ± 0.262	0.582 ± 0.243	0.560 ± 0.256

**Table 4:** Data distribution through activities [53].

Activity	Phone			Watch		Total	Class %
	Accel	Gyro		Accel	Gyro		
Walking	1271	936		1011	915	4133	5.5%
Jogging	1314	966		993	902	4175	5.6%
Stairs	1180	946		997	865	3988	5.3%
Sitting	1263	984		1028	939	4214	5.6%
Standing	1283	969		1046	934	4232	5.6%
Typing	1180	938		988	900	4006	5.3%
Brush Teeth	1282	954		1006	918	4160	5.5%
Eat Soup	1252	974		1012	899	4137	5.5%
Eat Chips	1236	947		1011	922	4116	5.5%
Eat Pasta	1179	959		978	911	4027	5.4%
Drinking	1310	976		1044	954	4284	5.7%
Eat Sandwich	1242	949		980	915	4086	5.4%
Kicking	1466	971		1009	919	4365	5.8%
Catch	1431	944		1015	903	4293	5.7%
Dribbling	1413	972		1027	939	4351	5.8%
Writing	1241	948		1038	948	4175	5.6%
Clapping	1270	978		1009	917	4174	5.6%
Fold Clothes	1261	970		1019	933	4183	5.6%
Total	23,074	17,281		18,211	16,533	75,099	100.0%

## Data Availability

Suggested data availability statements are available in Section 4.2.

## References

[1] World Health Organization (2022). Global Status Report on Physical Activity 2022.

[2] Sallis J.F., Bull F., Guthold R., Heath G.W., Inoue S., Kelly P., Oyeyemi A.L., Perez L.G., Richards J., Hallal P.C. (2016). Progress in physical activity over the Olympic quadrennium. Lancet.

[3] Cheatham S.W., Stull K.R., Fantigrassi M., Motel I. (2018). The efficacy of wearable activity tracking technology as part of a weight loss program: A systematic review. J. Sports Med. Phys. Fitness.

[4] Reeder B., David A. (2016). Health at hand: A systematic review of smart watch uses for health and wellness. J. Biomed. Inform..

[5] Foster K.R., Torous J. (2019). The opportunity and obstacles for smartwatches and wearable sensors. IEEE Pulse.

[6] Zhang S., Li Y., Zhang S., Shahabi F., Xia S., Deng Y., Alshurafa N. (2022). Deep learning in human activity recognition with wearable sensors: A review on advances. Sensors.

[7] Abdel-Salam R., Mostafa R., Hadhood M. (2021). Human activity recognition using wearable sensors: Review, challenges, evaluation benchmark. Deep Learning for Human Activity Recognition, Proceedings of the Second International Workshop, DL-HAR 2020, Held in Conjunction with IJCAI-PRICAI 2020, Proceedings 2, Kyoto, Japan, 8 January 2021.

[8] Vaizman Y., Ellis K., Lanckriet G. (2017). Recognizing detailed human context in the wild from smartphones and smartwatches. IEEE Pervasive Comput..

[9] Morris D., Saponas T.S., Guillory A., Kelner I. RecoFit: Using a wearable sensor to find, recognize, and count repetitive exercises. Proceedings of the SIGCHI Conference on Human Factors in Computing Systems.

[10] Anguita D., Ghio A., Oneto L., Parra X., Reyes-Ortiz J.L. (2013). A public domain dataset for human activity recognition using smartphones. Esann.

[11] Dasso N.A. (2019). How is exercise different from physical activity? A concept analysis. Nursing Forum.

[12] Ramanujam E., Perumal T., Padmavathi S. (2021). Human activity recognition with smartphone and wearable sensors using deep learning techniques: A review. IEEE Sens. J..

[13] Mohamed A., Lejarza F., Cahail S., Claudel C., Thomaz E. HAR-GCNN: Deep Graph CNNs for Human Activity Recognition From Highly Unlabeled Mobile Sensor Data. Proceedings of the 2022 IEEE International Conference on Pervasive Computing and Communications Workshops and Other Affiliated Events (PerCom Workshops).

[14] Chen L., Zhang Y., Miao S., Zhu S., Hu R., Peng L., Lv M. (2022). SALIENCE: An unsupervised user adaptation model for multiple wearable sensors based human activity recognition. IEEE Trans. Mob. Comput..

[15] Cruciani F., Vafeiadis A., Nugent C., Cleland I., McCullagh P., Votis K., Giakoumis D., Tzovaras D., Chen L., Hamzaoui R. (2020). Feature learning for human activity recognition using convolutional neural networks: A case study for inertial measurement unit and audio data. CCF Trans. Pervasive Comput. Interact..

[16] Nutter M., Crawford C.H., Ortiz J. Design of novel deep learning models for real-time human activity recognition with mobile phones. Proceedings of the 2018 International Joint Conference on Neural Networks (IJCNN).

[17] Zhu R., Xiao Z., Li Y., Yang M., Tan Y., Zhou L., Lin S., Wen H. (2019). Efficient human activity recognition solving the confusing activities via deep ensemble learning. IEEE Access.

[18] Dua N., Singh S.N., Semwal V.B. (2021). Multi-input CNN-GRU based human activity recognition using wearable sensors. Computing.

[19] Challa S.K., Kumar A., Semwal V.B. (2022). A multibranch CNN-BiLSTM model for human activity recognition using wearable sensor data. Vis. Comput..

[20] Mutegeki R., Han D.S. A CNN-LSTM approach to human activity recognition. Proceedings of the 2020 International Conference on Artificial Intelligence in Information and Communication (ICAIIC).

[21] Zhang X., Zhao J., LeCun Y. (2015). Character-level convolutional networks for text classification. Adv. Neural Inf. Process. Syst..

[22] Bai S., Kolter J.Z., Koltun V. (2018). An empirical evaluation of generic convolutional and recurrent networks for sequence modeling. arXiv.

[23] Singh S.P., Sharma M.K., Lay-Ekuakille A., Gangwar D., Gupta S. (2020). Deep ConvLSTM with self-attention for human activity decoding using wearable sensors. IEEE Sens. J..

[24] Ordóñez F.J., Roggen D. (2016). Deep convolutional and lstm recurrent neural networks for multimodal wearable activity recognition. Sensors.

[25] Bock M., Hölzemann A., Moeller M., Van Laerhoven K. Improving deep learning for HAR with shallow LSTMs. Proceedings of the 2021 International Symposium on Wearable Computers.

[26] Mahmud S., Tonmoy M., Bhaumik K.K., Rahman A.M., Amin M.A., Shoyaib M., Khan M.A.H., Ali A.A. (2020). Human activity recognition from wearable sensor data using self-attention. arXiv.

[27] Kuncan F., Kaya Y., Yiner Z., Kaya M. (2022). A new approach for physical human activity recognition from sensor signals based on motif patterns and long-short term memory. Biomed. Signal Process. Control.

[28] Ponsam J.G., Gracia S.J.B., Geetha G., Nimala K., Chepuri S., Rajline R.S. Human Activity Recognition Using LSTM Network with Dropout Technique. Proceedings of the 2022 International Conference on Power, Energy, Control and Transmission Systems (ICPECTS).

[29] Khatun M.A., Yousuf M.A., Ahmed S., Uddin M.Z., Alyami S.A., Al-Ashhab S., Akhdar H.F., Khan A., Azad A., Moni M.A. (2022). Deep CNN-LSTM with self-attention model for human activity recognition using wearable sensor. IEEE J. Transl. Eng. Health Med..

[30] Thakur D., Biswas S., Ho E.S., Chattopadhyay S. (2022). Convae-lstm: Convolutional autoencoder long short-term memory network for smartphone-based human activity recognition. IEEE Access.

[31] Mekruksavanich S., Jantawong P., Hnoohom N., Jitpattanakul A. Refined LSTM Network for Sensor-based Human Activity Recognition in Real World Scenario. Proceedings of the 2022 IEEE 13th International Conference on Software Engineering and Service Science (ICSESS).

[32] Murthy R., Dhanraj S., Manjunath T., Achyutha P., Prasad A., Gangambika G. (2022). A survey on human activity recognition using CNN and LSTM. Int. J. Health Sci..

[33] Jordao A., Nazare Jr A.C., Sena J., Schwartz W.R. (2018). Human activity recognition based on wearable sensor data: A standardization of the state-of-the-art. arXiv.

[34] Banos O., Garcia R., Holgado-Terriza J.A., Damas M., Pomares H., Rojas I., Saez A., Villalonga C. (2014). mHealthDroid: A novel framework for agile development of mobile health applications. Ambient Assisted Living and Daily Activities, Proceedings of the 6th International Work-Conference, IWAAL 2014, Belfast, UK, 2–5 December 2014.

[35] Zhang M., Sawchuk A.A. USC-HAD: A daily activity dataset for ubiquitous activity recognition using wearable sensors. Proceedings of the 2012 ACM Conference on Ubiquitous Computing.

[36] Chen C., Jafari R., Kehtarnavaz N. UTD-MHAD: A multimodal dataset for human action recognition utilizing a depth camera and a wearable inertial sensor. Proceedings of the 2015 IEEE International conference on Image PROCESSING (ICIP).

[37] Bruno B., Mastrogiovanni F., Sgorbissa A. A public domain dataset for ADL recognition using wrist-placed accelerometers. Proceedings of the the 23rd IEEE International Symposium on Robot and Human Interactive Communication.

[38] Weiss G.M., Yoneda K., Hayajneh T. (2019). Smartphone and smartwatch-based biometrics using activities of daily living. IEEE Access.

[39] Fazli M., Kowsari K., Gharavi E., Barnes L., Doryab A. (2020). HHAR-Net: Hierarchical human activity recognition using neural networks. arXiv.

[40] Tan Y.F., Poh S.C., Ooi C.P., Tan W.H. (2023). Human activity recognition with self-attention. Int. J. Electr. Comput. Eng. IJECE.

[41] Sztyler T. (2019). Sensor-Based Human Activity Recognition: Overcoming Issues in a Real World Setting.

[42] Stisen A., Blunck H., Bhattacharya S., Prentow T.S., Kjærgaard M.B., Dey A., Sonne T., Jensen M.M. Smart devices are different: Assessing and mitigatingmobile sensing heterogeneities for activity recognition. Proceedings of the 13th ACM Conference on Embedded Networked Sensor Systems.

[43] Garcia-Gonzalez D., Rivero D., Fernandez-Blanco E., Luaces M.R. (2020). A public domain dataset for real-life human activity recognition using smartphone sensors. Sensors.

[44] Roggen D., Calatroni A., Rossi M., Holleczek T., Förster K., Tröster G., Lukowicz P., Bannach D., Pirkl G., Ferscha A. Collecting complex activity datasets in highly rich networked sensor environments. Proceedings of the 2010 Seventh International Conference on Networked Sensing Systems (INSS).

[45] Scholl P.M., Wille M., Van Laerhoven K. Wearables in the wet lab: A laboratory system for capturing and guiding experiments. Proceedings of the 2015 ACM International Joint Conference on Pervasive and Ubiquitous Computing.

[46] Reyes-Ortiz J.L., Oneto L., Samà A., Parra X., Anguita D. (2016). Transition-aware human activity recognition using smartphones. Neurocomputing.

[47] Wang G., Li Q., Wang L., Wang W., Wu M., Liu T. (2018). Impact of sliding window length in indoor human motion modes and pose pattern recognition based on smartphone sensors. Sensors.

[48] Lara O.D., Labrador M.A. (2012). A survey on human activity recognition using wearable sensors. IEEE Commun. Surv. Tutorials.

[49] Sztyler T., Stuckenschmidt H. On-body localization of wearable devices: An investigation of position-aware activity recognition. Proceedings of the 2016 IEEE International Conference on Pervasive Computing and Communications (PerCom).

[50] Kunjan S., Grummett T.S., Pope K.J., Powers D.M., Fitzgibbon S.P., Bastiampillai T., Battersby M., Lewis T.W. (2021). The necessity of leave one subject out (LOSO) cross validation for EEG disease diagnosis. Brain Informatics, Proceedings of the 14th International Conference, BI 2021, Virtual Event, 17–19 September 2021.

[51] Kwapisz J.R., Weiss G.M., Moore S.A. (2011). Activity recognition using cell phone accelerometers. ACM SigKDD Explor. Newsl..

[52] Lane N.D., Bhattacharya S., Mathur A., Georgiev P., Forlivesi C., Kawsar F. (2017). Squeezing deep learning into mobile and embedded devices. IEEE Pervasive Comput..

[53] Weiss G.M. (2019). Wisdm smartphone and smartwatch activity and biometrics dataset. UCI Mach. Learn. Repos. Wisdm Smartphone Smartwatch Act. Biom. Dataset Data Set.

